# Your body, my body, our coupling moves our bodies

**DOI:** 10.3389/fnhum.2014.01004

**Published:** 2014-12-16

**Authors:** Guillaume Dumas, Julien Laroche, Alexandre Lehmann

**Affiliations:** ^1^Institut Pasteur, Human Genetics and Cognitive Functions UnitParis, France; ^2^CNRS UMR3571 Genes, Synapses and Cognition, Institut PasteurParis, France; ^3^Human Genetics and Cognitive Functions, University Paris Diderot, Sorbonne Paris CitéParis, France; ^4^Akoustic ArtsParis, France; ^5^Department of Otolaryngology - Head and Neck Surgery, Faculty of Medicine, McGill UniversityMontreal, QC, Canada; ^6^Centre for Research on Brain, Language and MusicMontreal, QC, Canada; ^7^International Laboratory for Brain, Music and Sound ResearchMontreal, QC, Canada

**Keywords:** coupling, coordination, interpersonal, dynamical systems, human interaction

Sensitivity to temporal contingencies appears early in life and plays a key role in the ontogeny of socio-cognitive abilities in humans (Nadel et al., 1999; Gratier and Apter-Danon, 2009). The tendency for rhythmic coordination, sometimes referred to as “entrainment,” requires sensory-motor coupling (Phillips-Silver et al., 2010). In most of the fields of cognitive science, action-perception and agent-world coupling views are replacing the classical stimulus-response dichotomy (Marsh et al., 2009; Silberstein and Chemero, 2012; Schilbach et al., 2013; Novembre and Keller, 2014). Such conceptual frameworks are well suited to study coordination phenomena as they emphasize the dynamical nature of cognition (Varela et al., 1993; Kelso, 1995; Buzsáki and Draguhn, 2004; Lehmann and Schönwiesner, 2014). Moreover, they leave room for the balance of autonomy, a central feature of complex biological systems, and interactive coupling, through which such systems relate to—and make sense of—their environment (Di Paolo, 2005; Barandiaran et al., 2009; Buhrmann et al., 2013). A naturalistic study of autonomy and coupling requires both embracing ecological situations and considering first-person perspective. Furthermore, many social coordination phenomena cannot be observed in the laboratory without the interaction of at least two subjects. We propose to consider linking first- and third-person measures, and even relate them across multiple interacting individuals. We will discuss how these concepts are intertwined in coordination phenomena, and outline existing methods to address those issues.

## Coupling and autonomy, the two faces of coordination

Coordination is related to two complementary aspects: autonomy and interactive coupling (Clayton et al., 2004). Autonomy refers to the intrinsic laws of organization of a living system (Varela, 1979). In turn, such laws define the environment to which a living system can couple. Autonomy thus provides a system with a dynamical background in the context of which sensory perturbations occur; it provides a perspective that shapes the lived world. In turn, as a result of their modulation, internal dynamics carry the imprint of the system's own environment. In other words, internal dynamics depend on agent~world relational dynamics (i.e., they depend on the way the relation between agent and world evolves). In short, complex systems and their environment co-determine each other. Patterns of coordination thus emerge dynamically from agent~world coupling and are therefore both autonomous and relational. By emergence, we mean that despite the fact that coordination is a by-product of intra-individual processes (Ross and Balasubramaniam, 2014), it is not reducible to them. In this opinion, we emphasize the inter-individual dimension of coordination, especially in the case of the human specific activities of music and dance [numerous ways of interacting have been studied in animals and humans (Strogatz, 2003), ranging from flock behavior (Okubo, 1986) to language (Dale et al., 2013; Manson et al., 2013)].

Rhythmic coordination of movements emerges from the dynamics of interaction between multiple component processes (Kelso, 1995; see also Van Orden et al., 2003); those interactions bring forth a dynamical landscape that pre-orients behavior and that can be modulated by intention (Kelso, 2002). For instance, rhythmic coordination of two limbs is driven by the dynamics of their relation, rather than by their sole intrinsic properties (Kelso, 1984). Coordination of one limb to an external pacer is governed by their relational dynamics as well (Kelso, 1981). Such dynamical interactions lead to coordination of activity across multiple scales of brain and behavior (Ihlen and Vereijken, 2010; Kelso et al., 2013). Human subjects can embody both pluri-frequential rhythms (Toiviainen et al., 2010) and the complexity of their fluctuations (Rankin et al., 2009; Marmelat et al., 2014). Thanks to the intrinsic complexity that underlies behavioral coordination, coupling allows coordination with the environment across scales (Laroche et al., 2014). If relational dynamics play a role in rhythmic coordination they may also play a key role in social interaction. Indeed, interacting with others is organized rhythmically and at multiple timescales since infancy (Gratier and Apter-Danon, 2009). The development of our capacity to coordinate rhythmically might lie in the dynamics of social interactions, rather than in purely intra-individual processes (Dumas, 2011): while we obviously need intra-individual capacities (e.g., vision) to handle social interaction, the development of our coordination capacities is shaped by those interactions as well (De Jaegher et al., 2010; Dumas et al., 2014b; Froese et al., 2014b)

Two-body approaches—at least two participants interacting in real-time—demonstrate that interactive contexts bring forth different qualities of coordination, in comparison with perceptive contexts in which participants' behaviors do not have any impact on their environment. Indeed, mutual interaction allows for more accurate/stable coordination than unilateral situations where only one partner is responsive to the other (Cummins, 2009; Konvalinka et al., 2010; Noy et al., 2011). Dynamics that are properly collective thus arise in mutual interaction, and they can attract and coordinate individual behaviors (De Jaegher and Di Paolo, 2007; Auvray et al., 2009; Lenay and Stewart, 2012; Laroche and Kaddouch, 2014). Such dynamics are therefore irreducible to purely intra-individual processes. Interacting subjects can yet rely on those relational dynamics; they can jointly regulate them. Overall, neither the dynamics of the interaction process nor their co-regulation can be observed when subjects are isolated from each other. It is thus important to study the dynamical properties of the interaction process itself in order to understand how we co-regulate them. More individually-centered processes of coordination might derive from recurrent social interactions. Recent methodological and technological advances make this change of paradigm possible.

## Deciphering factors enabling coordination through an ecological and dynamical approach

Recent technological improvements made the continuous tracking of complex movements possible. For instance, whole-body motion capture through multi-camera settings showed that subjects can coordinate their movements simultaneously to multiple timescales of musical events (Toiviainen et al., 2010). Low-cost motion capture devices such as wireless accelerometers from video-game devices have been used to ecologically investigate interpersonal coordination between listeners dancing to music (De Bruyn et al., 2009). Recent analytical tools can deal with non-linear dynamics of movements and interpersonal coordination across multiple timescales [e.g., windowed-cross correlation (Boker et al., 2002); cross-wavelet transform (Issartel et al., 2007); frame-differencing methods (Paxton and Dale, 2013); cross-recurrence quantification analysis (Coco and Dale, 2014; Demos et al., 2014); detrended cross-correlation analysis (Hennig, 2014); multifractal detrended fluctuation analysis (Bedia et al., 2014)]. Such behavioral measures can discriminate between roles (i.e., leader/follower, Sacheli et al., 2013), individual strategies of regulation of coupling (Fairhurst et al., 2014), types of personality (Schmidt et al., 1994) or can identify signatures of social disorders (Varlet et al., 2014). With tools grasping the complexity of movements, more ecological experiments are within reach.

Several innovations in brain-imaging methods can be readily applied to the study of rhythmic coordination in music and dance contexts. Through a careful design of control conditions, the use of ecological musical stimuli is possible in fMRI (Blood and Zatorre, 2001) as well as in EEG with the Steady-States Evoked Potential (SS-EP) technique. Traditional event-related potential approaches require numerous stimuli repetitions; hence stimuli durations are usually kept to a minimum. With the SS-EP technique, a continuous stimulus such as music that has a periodic structure (or is frequency-tagged) can be presented and requires very few repetitions. It has been used successfully to demonstrate neural oscillations underlying listening and tapping to synthetic beats (Nozaradan et al., 2011, 2012, 2013) and could be extended to study ecological musical beats. Adequately studying natural cognition may require the integration of multiple modality and sensing techniques, while participants move freely (Makeig et al., 2009; Gramann et al., 2014). The recent years have seen the development of wearable devices for electrophysiological (Codrons et al., 2014), as well as EEG recordings (Debener et al., 2012; De Vos et al., 2014), allowing to experiment in contexts more ecological than the laboratory (e.g., a concert venue). As smartphones get powerful enough to process brain signals in real-time, conducting in-field or at-home EEG protocols is becoming feasible (Stopczynski et al., 2014). Because those systems are low-cost and yet can provide research-grade quality signals (Badcock et al., 2013), they can easily scale up to record multiple participants.

Over the last decade, social neuroscience took an interactive turn. Two-body and second-person neuroscience have especially been supporting the use of ecological paradigms for understanding the neural underpinning of social interaction (Schilbach et al., 2013). This ongoing interactive turn relies on the development of new methods. Hyperscanning, for instance, allows recording the brain activity of multiple individuals engaged in an interaction. This approach already demonstrated differential effects of social context (e.g., induced/spontaneous, see Dumas et al., 2012a) and role (e.g., leader/follower, see Dumas et al., 2012a; Sänger et al., 2013; Konvalinka et al., 2014) during interpersonal coordination. Music is an ideal ecological context for the study of coordination and has been used in the burgeoning field of social interaction neuroscience. The work of Lindenberger and colleagues has for instance revealed the inter-individual brain dynamics of joint improvisation (Müller et al., 2013) and how the global system should be described through both intra- and inter-individual processes (Sänger et al., 2012). An open question is how much does the observed inter-brain relationships rely on shared biological structure (Dumas et al., 2012b), task and environment (Burgess, 2013), or even cultural background (Vogeley and Roepstorff, 2009; Kitayama and Park, 2010). For instance, heart rate coordination can be induced by a common task (e.g., singing the same song in Vickhoff et al., 2013) or socially modulated coupling (Konvalinka et al., 2011). Neurocomputational modeling has already helped to measure the potential contribution of similarity at both anatomical and dynamical levels to our propensity to coordinate with others (Dumas et al., 2012b). Such empirically grounded models can moreover be combined with experiments through human-machine interaction (Dumas et al., 2014a). This is especially interesting for operationalizing real time and reciprocal social interactions while keeping a rigorous experimental control (e.g., parametrically manipulate coupling).

While at the objective level, many approaches have been proposed to relate intra- and inter-individual dynamics (Hasson et al., 2012; Konvalinka and Roepstorff, 2012; Dumas et al., 2014b), the link between the third (objective) and first person (subjective) accounts remains unclear. How can interaction help escaping this dichotomy? We argue that closing this gap requires a joint study of intrinsic and relational dynamics. Introspection in experimental psychology has been heavily criticized in the past decades, but more rigorous approaches are now being designed to study subjective experience while overcoming previous limitations (Bockelman et al., 2013; Petitmengin and Lachaux, 2013). Recent work has managed to question the lived experience of the intersubjective dimension of coordination (Froese et al., 2014a). The neurophenomenological approach demonstrates the feasibility of integrating first-person data with objective measures from cognitive neuroscience (Lutz et al., 2002; Fox et al., 2013). Despite those promising advances, the joint study of first and third person perspectives remains underrepresented in the literature. Music can provide a nice entry point to bridge the gap between the intimate subjective experience and objective brain~body processes (Flaig and Large, 2014). For instance, listeners experience modulates both emotional and neural responses (Chapin et al., 2010) and expert dancers display greater coherence between their subjective and physiological aspects of emotion (Sze et al., 2010).

## Conclusion

We have seen how rhythmic and social coordination rely on both intrinsic and relational dynamics, namely autonomy and coupling. The complementarity of those two aspects of coordination has been empirically demonstrated, but many challenges remain. Two issues appear at reach with available methodologies: (1) quantifying how interactions with the environment and others have a causal role at the intra-individual level and non-additive consequences at the inter-individual level, and (2) deciphering the different factors of coupling across neural, behavioral and cultural scales. A last challenge is to embrace the ongoing change of paradigm that shall bridge the gap between the lived and the observed experience of social coordination in ecological contexts. Taken together, integrating first-, second- and third-person perspectives is a required move to accurately study natural human coordination phenomena.

### Conflict of interest statement

The authors declare that the research was conducted in the absence of any commercial or financial relationships that could be construed as a potential conflict of interest.
